# Identifications and classifications of human locomotion using Rayleigh-enhanced distributed fiber acoustic sensors with deep neural networks

**DOI:** 10.1038/s41598-020-77147-2

**Published:** 2020-12-03

**Authors:** Zhaoqiang Peng, Hongqiao Wen, Jianan Jian, Andrei Gribok, Mohan Wang, Sheng Huang, Hu Liu, Zhi-Hong Mao, Kevin P. Chen

**Affiliations:** 1 Department of Electrical and Computer Engineering, University of Pittsburgh, Pittsburgh, 15260 USA; 2 National Engineering Laboratory for Fiber Optic Sensing Technology, Wuhan University of Technology, Wuhan, 430070 China; 3 Idaho National Laboratory, Idaho Falls, 83415 USA; 4 School of Instrument Science and Opto-electronic Engineering, Beihang University, Beijing, 10091 China; 5 National Energy Technology Laboratory, Pittsburgh, 15236 USA

**Keywords:** Imaging and sensing, Computer science

## Abstract

This paper reports on the use of machine learning to delineate data harnessed by fiber-optic distributed acoustic sensors (DAS) using fiber with enhanced Rayleigh backscattering to recognize vibration events induced by human locomotion. The DAS used in this work is based on homodyne phase-sensitive optical time-domain reflectometry (φ-OTDR). The signal-to-noise ratio (SNR) of the DAS was enhanced using femtosecond laser-induced artificial Rayleigh scattering centers in single-mode fiber cores. Both supervised and unsupervised machine-learning algorithms were explored to identify people and specific events that produce acoustic signals. Using convolutional deep neural networks, the supervised machine learning scheme achieved over 76.25% accuracy in recognizing human identities. Conversely, the unsupervised machine learning scheme achieved over 77.65% accuracy in recognizing events and human identities through acoustic signals. Through integrated efforts on both sensor device innovation and machine learning data analytics, this paper shows that the DAS technique can be an effective security technology to detect and to identify highly similar acoustic events with high spatial resolution and high accuracies.

## Introduction

Large-scale facilities and critical infrastructures are currently monitored via the heavy use of security technology such as cameras that operate in the visible or mid-infrared wavelengths. The fact that most of these cameras are equipped with facial recognition software has raised privacy concerns globally. However, these devices only offer the limited information they gather through line of sight, and this information can be distorted by various disguises such as masks or heavy make-up. In addition to imaging technology, vibration-based sensor systems have also been widely used to monitor and detect various intrusion and trespassing events. Sensor networks constructed using electrical seismometers and accelerometers can be used to monitor and to spatially resolve location of vibration caused by human locomotion and other intrusion events. However, extensive deployment of point sensors such as camera or electrical vibration sensors requires significant installation costs, especially in large-scale infrastructures such as national borders, pipelines and railways, etc.

Over the last two decades, distributed fiber-optic sensors have been explored and deployed for structural health monitoring and prognoses. A unique trait of fiber-optic sensor technology is its application in distributed sensing. The optical fiber itself can be used as a sensor to perform, at high spatial resolution, both static and dynamic measurements of features such as temperature, strain, and acoustic detections. By exploiting various optical scattering mechanisms (i.e., Rayleigh, Raman, and Brillouin scattering) that naturally occur in optical fibers, distributed measurements can be achieved with a high spatial resolution of 1–10 m using optical fibers spanning up to 100 km in length.

Given the large-scale use of fiber-optic sensors in structural health monitoring, the same fiber-optic sensor network can be used in security monitoring. The inherent advantage of this lies in the fact that fiber-optic sensors are less invasive of personal privacy, less easily fooled by disguises and free from line-of-sight detection. Furthermore, installation of fiber-optic sensor networks has the potential to be significantly cheaper, as distributed fiber sensing schemes require only one or two pieces of optical fiber in order to cover a large facility. In the last two decades, several distributed or quasi-distributed fiber sensors were explored for security applications. Fiber sensors such as the Michelson interferometer^1^, Mach–Zehnder interferometer^2^, Sagnac interferometer^3^, fiber Bragg gratings (FBG) network^4^, Rayleigh optical time-domain reflectometry (OTDR)^5^, φ-OTDR^6^, and Brillouin OTDR^7^ have been explored for use in intrusion detection through detection of vibration induced by human locomotion. Quasi-distributed FBG sensor networks have been used extensively to spatially locate various intrusion events and maintain perimeter security. Through wavelength- or time-division multiplexing, intrusion events can be detected with high spatial resolution^8^. Using various pattern-recognition algorithms such as principal component analysis and K-Nearest Neighbor classifier, movements (e.g. people climbing, knocking, digging, or walking; even cats jumping), can be successfully detected and classified.

However, the applications of sensor networks to not only detect but also to identify human locomotion is far more challenging than identifying acoustic event with distinctly different acoustic characteristics demonstrated before. Acoustic signal patterns of human locomotion produced by different individuals are weak and only have subtle difference among each other. Successful identifications of individual using his or her locomotion acoustic pattern requires both high quality sensor data in term of high signal to noise ratio (SNR) and high spatial resolution, as well as robust data analytics algorithms.

Fiber optical Distributed Acoustic Sensor (DAS) based on Rayleigh scattering, is a promising fiber sensor technology used in distributed vibration detection. It uses unperturbed fibers to determine the location and transient waveforms of acoustic events^9^. Rayleigh scattering-based OTDR can detect acoustic emissions via a wide frequency bandwidth. A 128 km φ-OTDR sensor reportedly detected intrusions with 15 m spatial resolution^10^. Therefore, DAS is an ideal solution to detect acoustic events with high spatial resolutions. The performance of a φ-OTDR system, such as the sensing dynamic range and sensitivity, is governed by the system SNR, which is limited by weak Rayleigh back-scattering coefficient of an unmodified optical fiber^11^. Most of researchers address low cadence of sensor data through data analytics. Simple data processing techniques, such as moving average and wavelet transformation, have been used to enhance the quality of sensor signals^12,13^. These techniques are time-consuming, and will decrease the maximum detectable frequency of φ-OTDR systems. Multiscale wavelet decomposition^14^, Gaussian mixture models^15,16^, and Morphologic feature extraction^17^ have been used as pattern recognition (PR) tools. However, the fundamental issue of weak backscattering signals in telecom-grade optical fibers still limits the performance of fiber-optic DAS sensors. Poor distributed sensor performance therefore severely limit effectiveness of data analytics to recognize significantly different acoustic patterns, such as human and motor vehicle movement or safe excavator operations and accidental excavator contact with a gas pipeline^18^.

To address these challenges, this paper, for the first time to our best knowledge, presents an integrated approach to improve performance of distributed fiber optical acoustic sensors to identify highly similar acoustic events such as human locomotion through innovations at both sensor device levels and through machine learning data analytics. Using a femtosecond laser direct writing scheme, Rayleigh backscattering of optical fibers were enhanced over 35 dB. This fundamentally address poor SNR of DAS using unmodified optical fibers to achieve high-precision dynamic strain measurements. Through deep neural network machine learning data analytics, this paper shows that it is possible to detect and identify human locomotion using distributed fiber sensors with high accuracy.

## Methods

### Sensor fabrications

In a typical DAS system using an φ-OTDR scheme, time-resolved Rayleigh backscattering from unmodified telecom single mode fibers is used as a sensing signal to detect phase changes caused by vibrations. However, telecom grade optical fibers have an intrinsically weak Rayleigh scattering profile that severely limits SNR in DAS systems. To mitigate this problem, a deep ultra-violet (DUV) laser was used to produce weak FBG in photosensitized fibers using a phase mask approach^19,20^. Recently, blank DUV laser exposure was used to enhance the Rayleigh scattering profile of telecom fibers^21^. Enhancement of up to 15 dB in the Rayleigh scattering profile is achievable through DUV radiation. However, both approaches require photosensitization of telecom fibers using a process such as hydrogen loading. Weak FBG and Rayleigh enhancement via DUV laser are not stable at elevated temperatures^22^.

**Figure 1:**
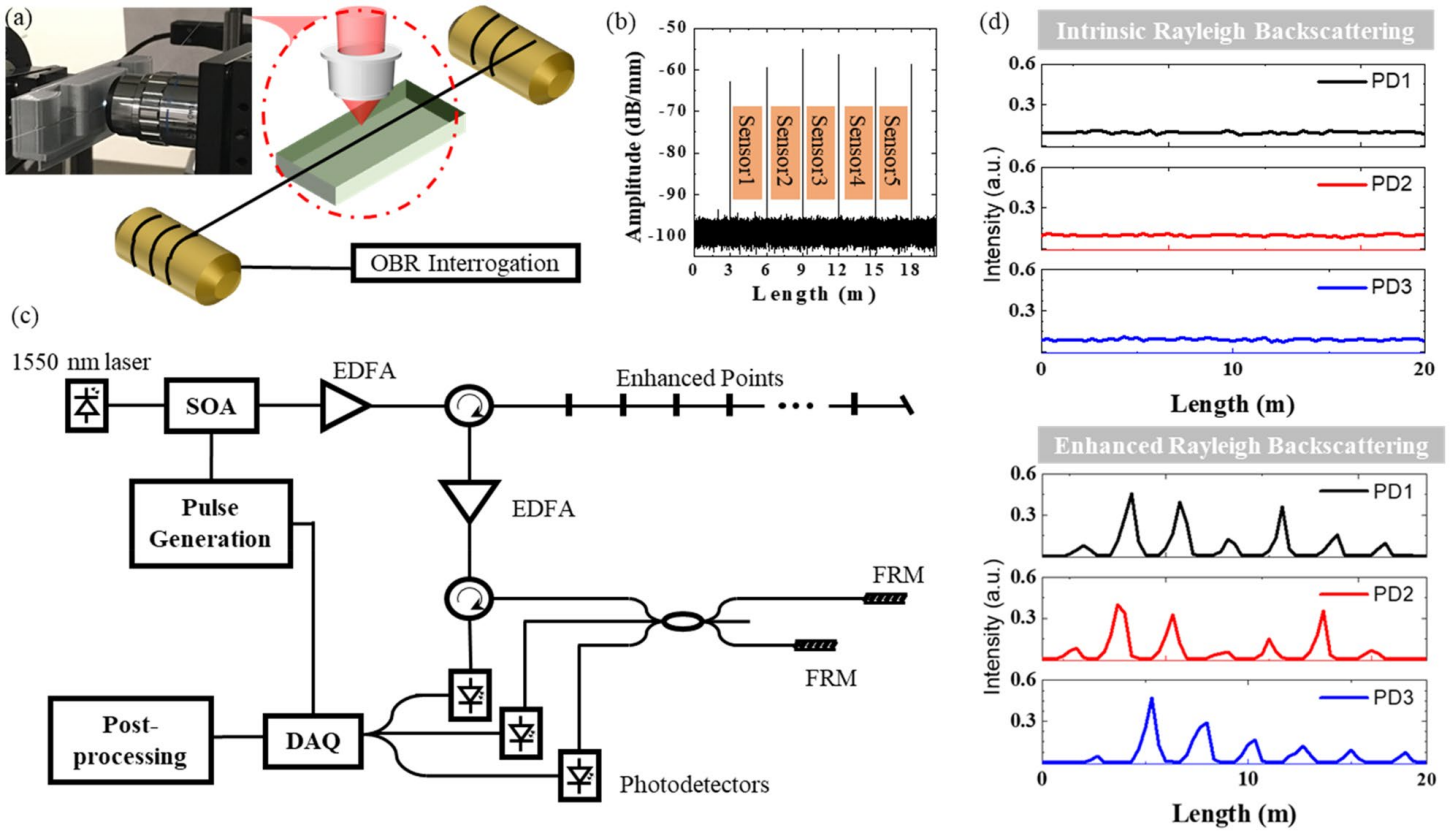
Enhanced backscattering by embedding nano-reflectors into sensing fiber. (**a**) Roll-to-roll setup of ultrafast laser direct writing. (**b**) Six nano-reflectors with Rayleigh enhancement of over 35 dB along 21 m of fiber. (**c**) Schematic diagram of the homodyne φ-OTDR sensing system inscribed with nano-reflectors. (**d**) Temporal profiles of intrinsic and enhanced Rayleigh backscattering signal detected by three photodetectors (PD1–3). (SOA: semiconductor optical amplifier; EDFA: erbium-doped fiber amplifier; DAQ: data acquisition; FRMs: Faraday rotation mirrors).

To address these challenges, an ultrafast laser roll-to-roll direct writing approach was used to induce temperature-stable artificial scattering centers as nano-reflectors for enhancing Rayleigh scattering. The detailed manufacturing process (see Fig. 1) can be found in a previous report^23^. The femtosecond laser system used for laser fabrication includes a Coherent Mira 900 mode-locked Ti:Sapphire oscillator and a Coherent RegA 9000 regenerative amplifier. The laser system generated 270-fs (FWHM) laser pulses at 800 nm with a 250 kHz repetition rate. By tightly focusing the ultrafast laser on the optic fiber core and using an oil-immersion objective (NA 1.25), nano-reflectors were inscribed at an on-target pulse energy of ~ 160 nJ. The nano-reflectors were inscribed through the protective jacket to preserve the fiber’s mechanical integrity. The irradiation time and laser pulse power were carefully adjusted for optimally enhanced level and low insertion loss. The detail laser inscription method and laser processing parameter optimization can be found in ref.^24^. The insertion loss of each Rayleigh-enhanced point went up following an increase in pulse energy when inscribing severe nano-defects. The optimal laser processing condition can produce nano-reflectors in sensing fiber with insertion loss at 0.0012-dB/reflector^24^.

Fabrication of weak reflectors in optical fibers does introduce additional attenuation, which could reduce the total sensing length. However, we have demonstrated that through optimization of the femtosecond laser direct writing process, insertion loss of each nano-reflectors can be minimized to 0.0012 dB/point. For example, a 2.5-km long sensing fiber with 830 nano-reflector points (~ 3-m spacing), will incur 1-dB additional insertion loss. This is a small penalty to pay given a 35 dB backscattering enhancement produced by nano-reflectors. This backscattering enhancement and insertion loss trade-off have been validated by previous works using FBGs as weak reflectors^25^.

Figure 1a shows the setup for nano-reflectors fabrication. A stand of telecom single mode fiber (Corning SMF-28e+) was translated via a roll-to-roll setup. The backscattering profile of the optical fiber was monitored by an Optical Backscatter Reflectometer (Luna OBR4600). The femtosecond laser inscribed, at 3-m intervals, six Rayleigh-enhanced points onto a fiber core spanning over 21 m. Each Rayleigh-enhanced point was 2 × 2-μm (cross section) by 7-μm (L) in the middle of the fiber core. Enhancements of over 35 dB were achieved through the laser direct writing process, as shown in Fig. 1b. Figure 1c shows the φ-OTDR distributed sensor interrogation setup. Unlike weak FBGs, which only reflect light at the FBG wavelength and are sensitive to temperature fluctuation, femtosecond laser-induced nano-reflectors produces broadband Rayleigh backscattering. The increased Rayleigh backscattering signal dramatically improves SNR. This is shown in Fig. 1d, while signals from three Photo detectors (PD1–PD3) from pristine fibers and those from Rayleigh enhanced fibers are compared. The Rayleigh enhanced backscattering points significantly improve SNR and thus precision of measurements.

### DAS phase demodulation

A schematic of the DAS system is shown in Fig. 1c. A DFB fiber laser with a 6 kHz linewidth and 1550.12 nm output wavelength (NKT Basik C15) was used as the interrogation laser source. The laser was modulated by a semiconductor optical amplifier (SOA, Thorlabs, SOA1013SXS) to generate a 15-ns pulse with a 50 kHz repetition rate. The laser pulses were amplified by an Erbium-doped fiber amplifier to reach 150-nJ/pulse (10 mW output power) and launched into the laser-enhanced fiber (fiber under test). The 3-meter sections of fiber between adjacent Rayleigh-enhanced points formed a fiber acoustic sensor for the DAS system. Therefore, five acoustic sensors were interrogated over a 21-m fiber span. The backscattered optical pulses were redirected to a 3 × 3 coupler through an optical circulator. The backscattered pulses were equally divided into 3 parts, as shown in Fig. 1c. Two output ports of the 3 × 3 coupler were connected to Faraday rotator mirrors. The optical path difference between the two output ports was tailored to be 3 m, matching the sensing fiber length between adjacent Rayleigh-enhanced points. Vibration-induced physical perturbation along the sensing fiber was then demodulated by three photodetectors (InGaAs Fixed Gain 200 MHz) using the Naval Postgraduate School (NPS) method^26^.

The relationship between the mechanical strain and refractive index in sensing fiber was described by the photo-elastic effect that determined the phase shift of transmitting light. The NPS demodulation method for phase extraction is briefly described in Eqs. (1) and (2). The three output signals of the 3 × 3 coupler have a 120° constant phase shift, which can be described as:

1 $$\left[\begin{array}{c}{V}_{out1}\\ {V}_{out2}\\ {V}_{out3}\end{array}\right]= \left[\begin{array}{l}C+Bcos\phi \left(t\right)\\ C+Bcos\left[\phi \left(t\right)-\frac{2}{3}\pi \right]\\ C+Bcos\left[\phi \left(t\right)+\frac{2}{3}\pi \right]\end{array}\right]$$

where $\phi \left(t\right)$ is the signal phase, $C$ is the DC component of voltage output from photodetectors, *B* is AC amplitude of photodetector’s voltage signals^27^. The phase of signal can be decomposed to $\phi \left(t\right)=\varphi \left(t\right)+\psi \left(t\right)$, where $\varphi \left(t\right)$ and $\psi \left(t\right)$ are phase shifts caused by physical perturbation and environmental noise, respectively. After some differential and integral operations, the output of the NPS algorithm is proportional to the value of $\phi \left(t\right)$:

2 $$Vout=\sqrt{3}A\phi \left(t\right)=\sqrt{3}A[\varphi \left(t\right)+\psi \left(t\right)]$$

where $A$ is the gain of operators in the NPS method. Therefore, phase changes caused by vibrations can be quantitatively detected for further analysis. More technical details on the DAS system using the 3 × 3 coupler demodulation scheme can be found in Ref.^25^.

### Experimental setup

**Figure 2:**
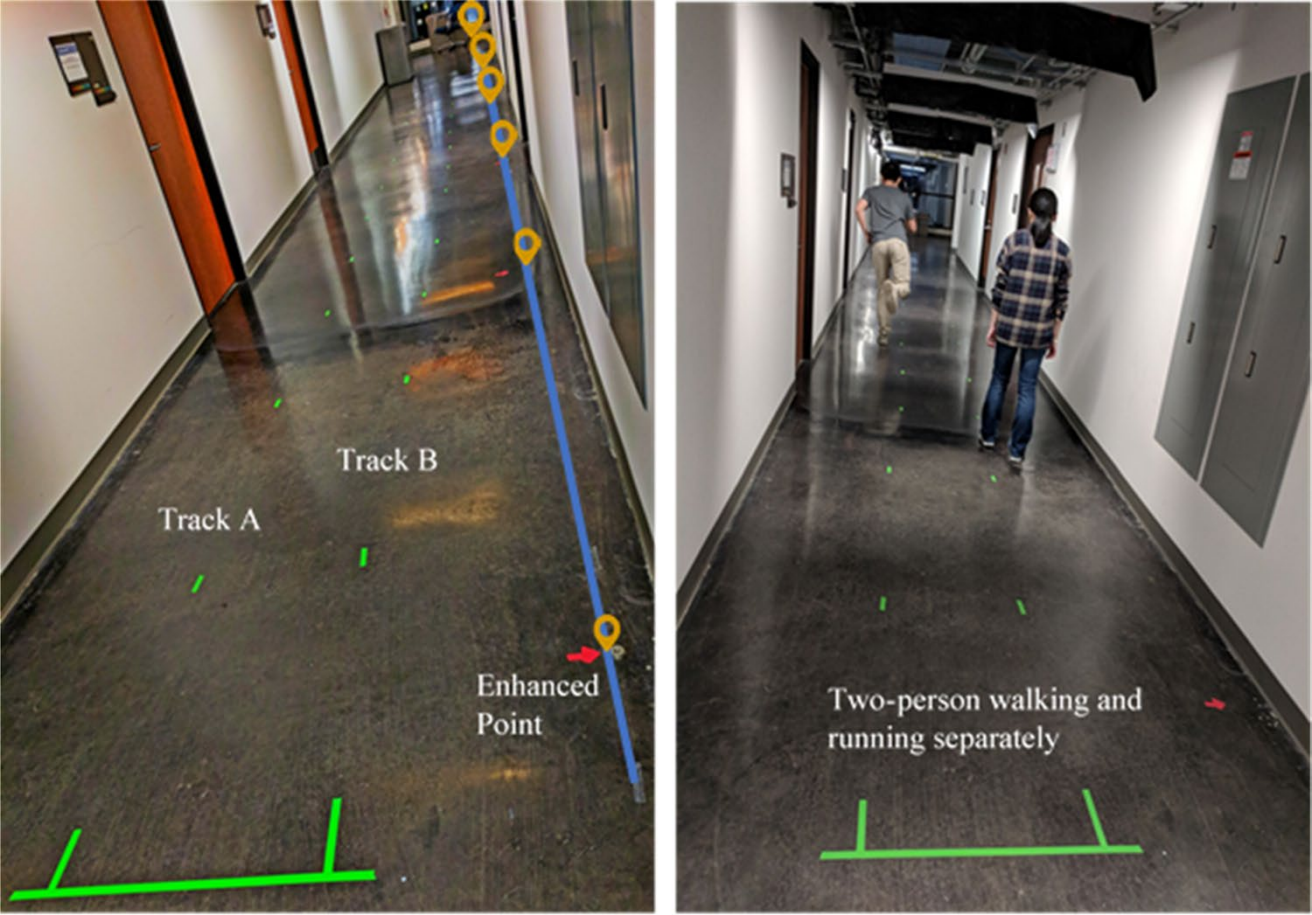
The test site with marks for two tracks and distributed fiber sensors on the ground. The example shows a simultaneous event featuring one person running and another walking.

Experiments were carried out in a 15-m-long hallway, shown in Fig. 2. The distributed fiber sensors were laid in a straight line on the concrete floor. Masking tape was used to introduce mechanic contact between the concrete floor and the sensors. Six Rayleigh backscattering-enhanced points were fabricated at 3-m intervals by an ultrafast laser to form five acoustic sensors for measuring floor vibrations. Two tracks were marked out on the concrete floor using green tape (see Fig. 2). The two tracks’ distance to the optical fiber was 80 cm and 140 cm, respectively.

### Data collection and pre-processing

Pedestrian identification was reported previously based on electronic vibration point sensors. Data analytic approach such as support vector machine (SVM)^28^ and iterative transductive learning algorithm^29^ have been used to delineate acoustic signal produced by solo walking pedestrian. Using distributed fiber sensors, a great diversity of locomotion data was harnessed in our experiments. These include acoustic signals produced by both walking and running persons (solo and pairs), and signals differentiated in human locomotion at different tracks, shoes and pushing cart. Multiple acoustic events induced by eight individuals were listed in Table 1. Both shallow and deep neural network machine learning algorithm were used to analyze and to classify sensor data. For acoustic events produced by a solo person, each participant repeated a given event (e.g., running or walking) 26 times on both tracks. Additional variations, such as acoustic signals generated by the same individual but wearing different shoes, were also recorded. Both human locomotion and mechanical system like pushing a cart was introduced as extra noise in order to test the robustness of the algorithm. For events involving two participants, random combinations of the individuals running and/or walking were selected.

**Table 1:** Description of dataset from events involving human movements.

Event	Number of participants	Number of tracks	Number of repetitions	Number of events
One person walking	8	2	26	416
One person walking with different shoe	1	1	26	26
One person walking with a cart	1	1	26	26
One person running	8	2	26	416
One person running with different shoe	1	1	26	26
Two people walking	4	1	26	104
Two people running	3	1	26	78
One person walking and another running	4	1	26	104
Total events	1196			

The multiple sensors and high bandwidth data acquisition led to an enormous amount of data. In total, acoustic signals generated by eight different events were recorded, producing 1196 sets of acoustic signals for machine learning application. The bandwidth for acoustic signals captured by the data acquisition system was capped at 2-kHz for each sensor (4-kHz sampling rate at 14 bits). Each sensor continually recorded a 1-s acoustic signal for ten sequential frames. The idle time between segmented frames was due to limited operational capability in the computer-based data acquisition and processing. Therefore, for each acoustic event, 200 k data points were recorded (4 k sampling/s × 5 sensors × 10 s).

**Figure 3:**
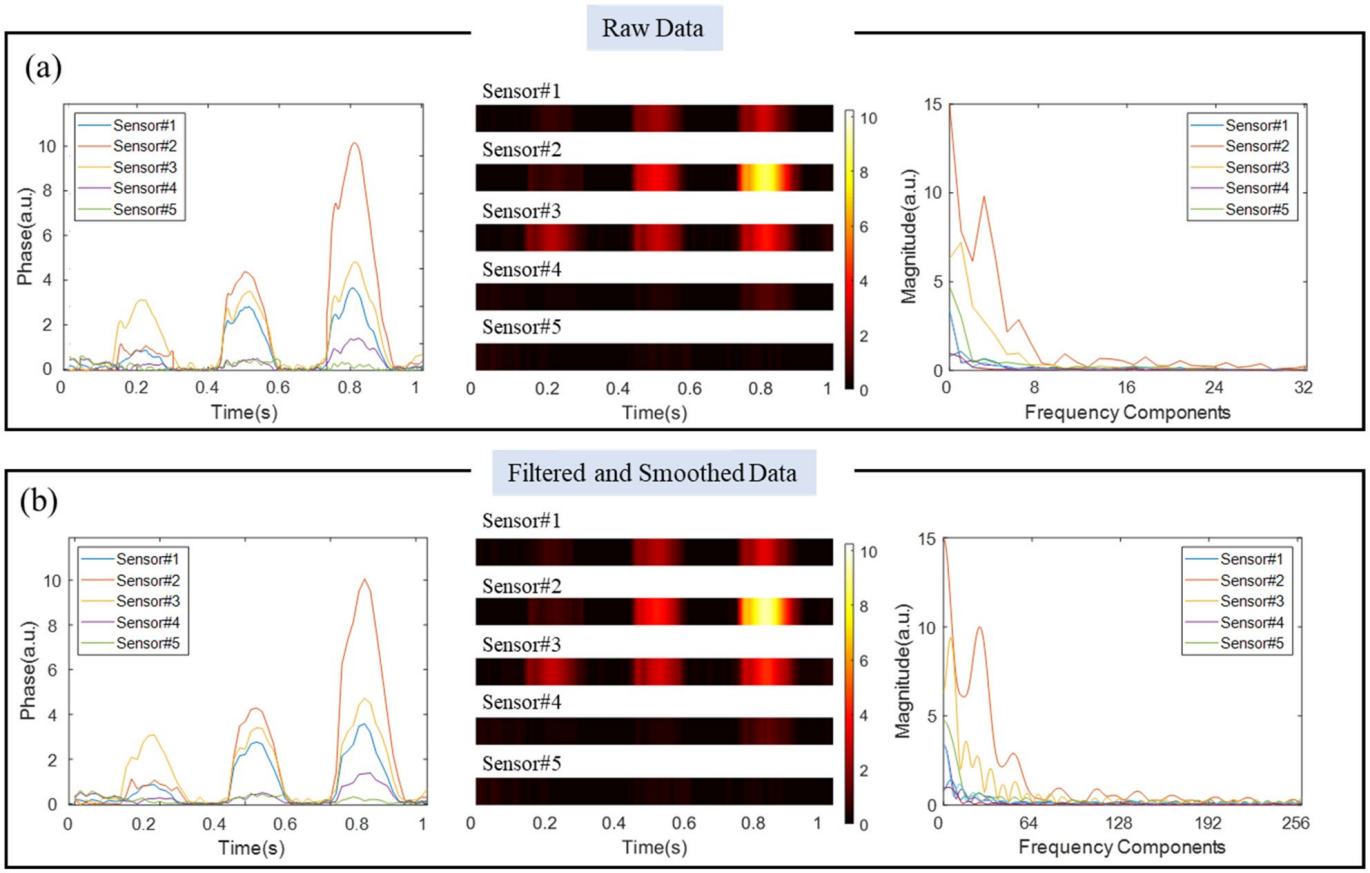
(**a**) Raw acoustic signals in temporal and frequency domain; and (**b**) acoustic signals after data preprocessing. Acoustic signals harnessed from all five sensors are overlaid together. Temporal data shown in (**a**) and (**b**) are also visualized using intensity maps.

Before applying machine learning methods for classification, the data were preprocessed to reduce computation times for the various machine learning algorithms. First, all acoustic data was transformed to frequency domains by Fast Fourier Transform (FFT) in order to offer neural networks direct access to the global properties of the signals. Figure 3a shows an example of raw acoustic signal harnessed by fiber sensors and its first 256 frequency components after FFT. Both our results shown in Fig. 3a and past studies have shown that human locomotion produce low-frequency vibration signals^30^. Therefore, a non-causal low-pass filter was applied to the signal in order to reduce the computational burden of the subsequent algorithm without losing relevant information. The order of a non-causal filter is infinite and the cutoff frequency is 32 Hz, which corresponds to the first 32 frequency components retained in each spectrum. A sinc filter was then used to smoothly interpolate these 32 amplitudes to 256 points, facilitating the deep learning algorithm shown below. As a result, the experimental data for an acoustic event (e.g., walking or running) consists of 1280 data points, which is 15 times less than the original dataset. The preprocessed data was arranged into a 3D format of 256 (frequency dimension) × 10  (s) × 5 (sensors). Figure 3b show the filtered and smoothed acoustic signal in both temporal and frequency domains.

Figure 3 shows both overlay curves plot of acoustic data and stacked intensity maps for visualization. It is evident that the data preprocessing using the low-pass filter keeps the predominant features of signals. Time-domain data presented in Fig. 3 shows simultaneous measurements of five sensors in response to the running motion of a single individual. It is easy to deduce that 3 foot-strikes occurred during this 1-s segment measurement. Given that Sensor 4 and 5 measured weak vibration signals, the foot-strikes of the runner occurred between the 0 and 9-m section of the hallway from the location of the first enhanced point. This is consistent with the actual event.

### Disclaimer

This work was funded by the Department of Energy, National Energy Technology Laboratory, an agency of the United States Government, through a support contract with Leidos Research Support Team (LRST). Neither the United States Government nor any agency thereof, nor any of their employees, nor LRST, nor any of their employees, makes any warranty, expressed or implied, or assumes any legal liability or responsibility for the accuracy, completeness, or usefulness of any information, apparatus, product, or process disclosed, or represents that its use would not infringe privately owned rights. Reference herein to any specific commercial product, process, or service by trade name, trademark, manufacturer, or otherwise, does not necessarily constitute or imply its endorsement, recommendation, or favoring by the United States Government or any agency thereof. The views and opinions of authors expressed herein do not necessarily state or reflect those of the United States Government or any agency thereof.

## Results

### Sensor data

**Figure 4:**
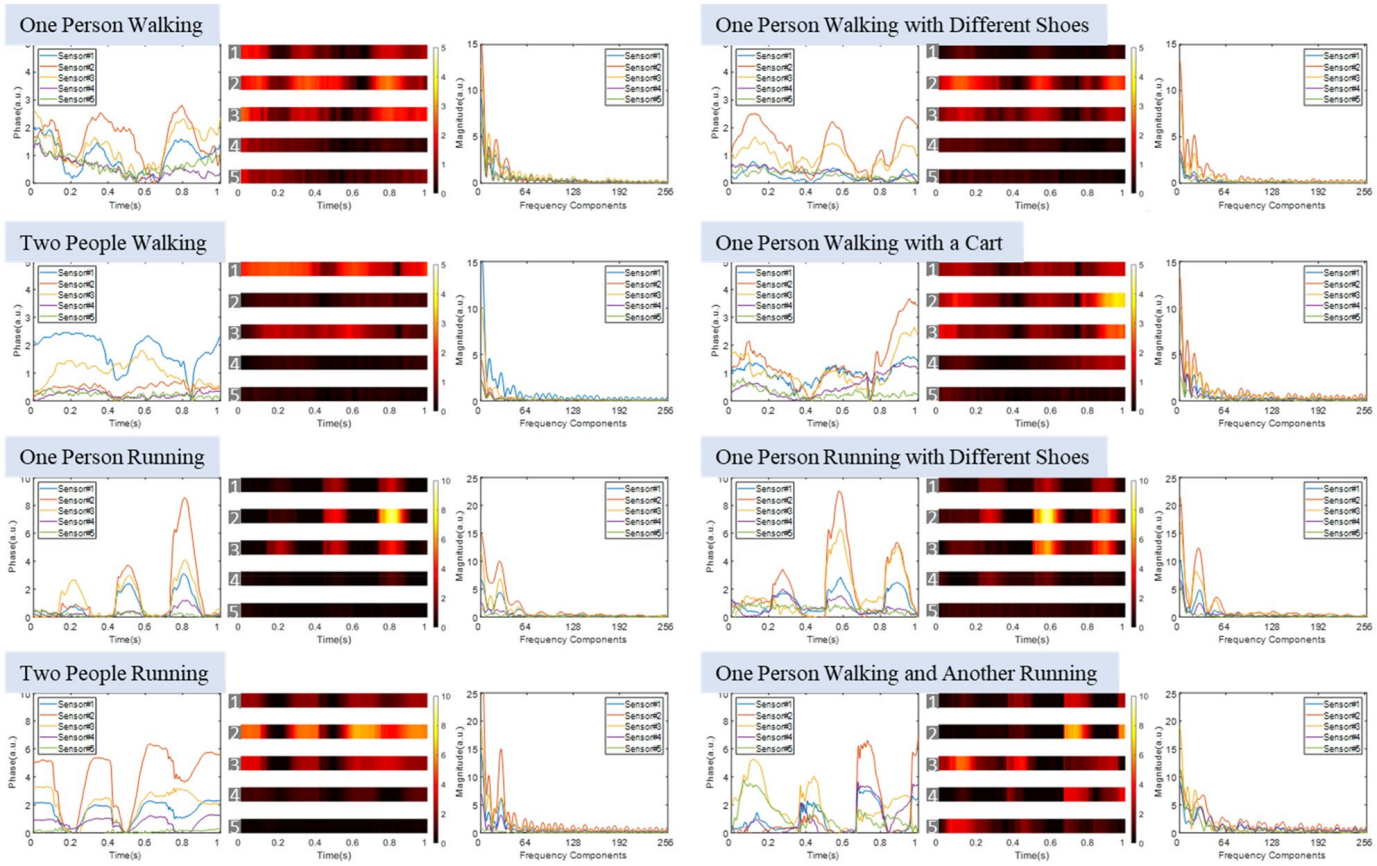
Plots of 1-s time-domain acoustic signals from five sensors are illustrated as overlaid curve plots and stacked intensity maps for visualization. The spectrum of first 256 frequency components after preprocessing are presented as well.

Variations in vibration intensity of the foot strikes also imply such things as running direction and/or imbalance in a person’s stride. These are cues for identifying the runner. However, identification through data analysis using the qualitative “eye-ball” approach in the time domain, or quantitative analysis in the frequency domain, become extremely difficult if a large number of participants or a wide range of events are involved. This challenge is shown in Fig. 4, which presents sensor data from various events, including walking or running with different footwear, walking while pushing a cart, and locomotion involving multiple participants. To highlight the frequency features of various events, only first 256 frequency components from vibration data in frequency domain is presented in Fig. 4. This is consistent with the vibration feature generated by human locomotive motions.

### Supervised machine learnings

Vibration-related information collected by the distributed fiber sensors includes high-frequency signals simultaneously harnessed from many locations. The enormous volume of data demands powerful analysis tools for extracting the covert features of specific individuals or acoustic events. Identification of a person or event through acoustic data relies on the extraction of subtle, complex patterns, such as the frequency/rhythm of human locomotion, imbalances in a person’s stride, and the temporal characteristics of foot impact (see Fig. 4). Though it is difficult to delineate these features using predictive data analyses, a machine learning approach could potentially handle the large amount of data from DAS and unveil patterns associated with specific individuals or acoustic events. The indoor pedestrian identification was reported previously based on electrical vibration sensors to classify slow-walking person by using SVM^28^ and iterative transductive learning algorithm^29^ . For this paper, a great diversity of locomotion data including walking and running at different tracks, shoes and pushing cart were harnessed by distributed optical fiber sensor. Both of supervised and unsupervised machine learnings were employed to analyze data in both the time and frequency domains. They included machine learning algorithms like SVM-based error-correcting output codes, multiple-hidden-layer convolutional neural network and unsupervised adaptive clustering.

#### Convolutional neural network (CNN)

**Figure 5:**
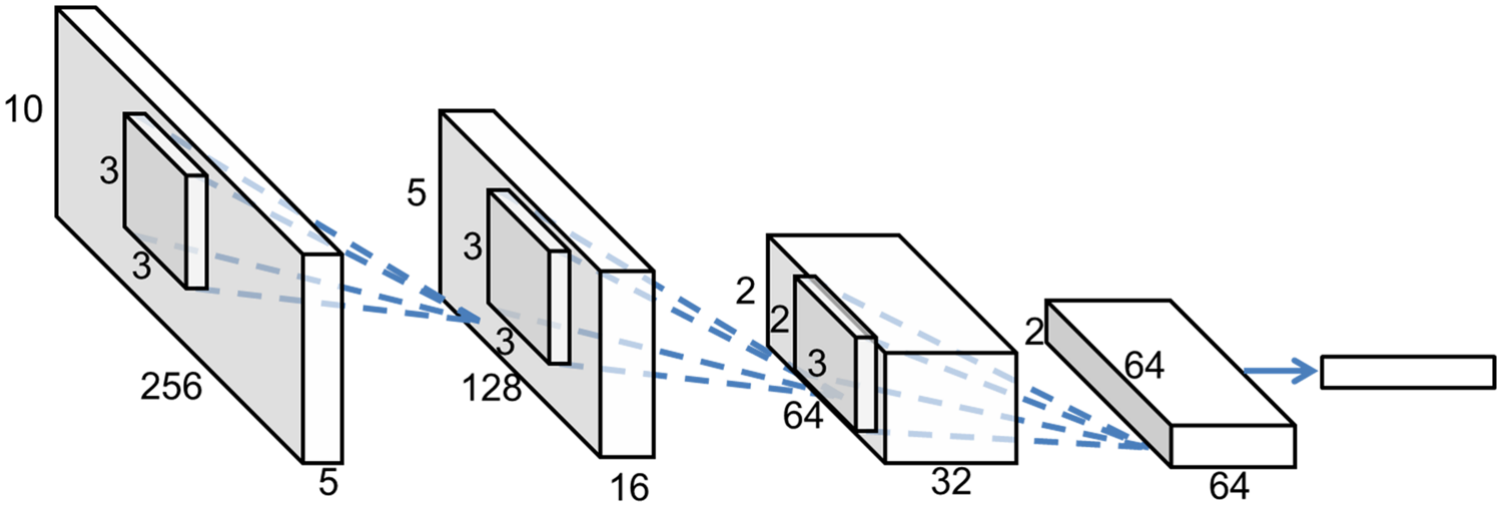
Architecture of the CNN.

CNN is a well-known machine learning algorithm that uses PR to identify images and various one-dimensional data. Its multiple layers of convolution filters extract local features of the input data and map them onto various categories of classification. During the non-linear activation and pooling processes, covert characteristics of acoustic events can be manifested and interpreted at the end of a fully connected layer. The acoustic signal harnessed from each sensor at a 1-s time frame was organized into a one-dimensional data sequence. Data acquired sequentially over ten 1-s time frames were arranged into a 2D data image. These 2D data images were then compiled into 3D data structures by stacking data from multiple sensors. By transforming into a 3D tensor within the frequency domain, the global properties of various intrusions can be efficiently accessed by CNN. Figure 5 shows the CNN structure used to identify movement.

The temporal acoustic signals were filtered and smoothed to become 256 frequency components. Ten 1-s frames and five sensors carried out in our experiment increased this vector to a 3D array as 256 (frequency components) × 10  (s) × 5 (sensors), which was fed into CNN.

Three layers of convolutional stages were applied in the CNN structure including 2 × 3 or 3 × 3 convolutional filters and 2 × 2 max pooling. The nonlinear activation function was Rectified Linear Unit, and max pooling was used between each layer to reduce data in each channel. Each layer of a CNN has two goals: increase the size of extracted features and reduce the size of raw data, so that the entire network transforms large size raw data into only useful features. The first goal is achieved through having increasing number of channels from one layer to the next. Each channel represents a feature and each unit in the channel decide whether the feature is present at that layer. The second goal is achieved through pooling. Pooling is similar to the process when an image being zoomed out. Nearby units compare their outputs and keep only one of them. In maxpooling, the one output they keep is the greatest output among them, so that the presence of the feature at that location can be passed onto the next layer. After each layer, the increase of channel numbers, which implied more features, were arranged and extracted. Based on those features, the output stage was set to be a fully connected softmax predicting the probability of fitting into different categories. Softmax is a function to map the non-normalized output of a network to a probability distribution over predicted output classes. It is the standard layer to transform the latent features extracted by the previous convolutional layers into the final classification result. Performance of the CNN was gauged for supervised machine learning, while various acoustic data generated by eight different events (Table 1) were used to train the CNN.

The recognition goals were set to be individuals, tracks, and shoes when using corresponding dataset for training. Acoustic signal dataset generated by various locomotion events summarized in Table 1 were partitioned into training dataset and testing dataset. For example, among 208 datasets of eight participants’ solo walking events, one hundred and twenty-eight datasets were randomly selected for training, while the rest were used for testing. Table 2 details number of datasets used for training and for testing, respectively. The acoustic signals generated by solo participants (running or walking) were also used to train neural networks to classify acoustic signals generated by two participants. Each algorithm was run ten times in order to obtain the range of uncertainty. To describe the prediction outcome as interval might be more informative when the lower limit of classification accuracy indicates the worse-case performance usually referring to the false alarms rate of system.

**Table 2:** Classification results using CNN for different scenarios.

Training	Testing	Recognition	Accuracy
One person walking (128)	One person walking (80)	Person	78.75–86.25%
One person walking on different tracks (32)	One person walking on different tracks (20)	Track	85.00–100.00%
One person walking with different shoes (32)	One person walking with different shoes (20)	Shoes	80.00–100.00%
One person walking and pushing a cart (32)	One person walking and pushing a cart (20)	Cart	93.75–100.00%
One person running (128)	One person running (80)	Person	76.25–86.25%
One person running on different tracks (32)	One person running on different tracks (20)	Track	95.00–100.00%
One person running with different shoes (32)	One person running with different shoes (20)	Shoes	75.00–90.00%
One person walking (208)	Two people walking (104)	Person	63.85–78.46% (either) 28.46–44.62% (both)
One person running (208)	Two people running (78)	Person	60.26–76.92% (either) 34.62–50.00% (both)
One person walking + one person running (416)	One person walking and another running (104)	Person and movement	54.62–69.23% (either) 46.92–61.54% (both)

The number of datasets for training and testing, respectively, is included in parentheses.

For datasets generated by a single person walking, CNN achieved greater than 78.75% accuracy in specifically identifying the individual (out of eight possibilities) who generated the acoustic signature. CNN achieved even greater accuracy (> 85%) in identifying the specific track(s) used by an individual. When the acoustic signal was generated by simultaneous walking and cart-pushing, CNN achieved better than 93% accuracy in identifying the event, thanks to the presence of the cart. CNN failed to identify the specific individual when different shoes were worn. However, when this dataset was singled out to train CNN, the neural network achieved over 80% accuracy in classifying the signals generated from different pairs of shoes.

Each time, the classifier achieved an accuracy of around 76.25% or higher in identifying the person making the movements, and over 80% accuracy in recognizing carts, tracks, and shoes, with no major difference in regard to walking or running. However, when the classifier was used to identify people in a two-person (grouped) activity, its accuracy decreased to 60.26% in identifying either person, and 28.46% in identifying both. In addition to a person’s identity, the classifier was used to identify movements, generating > 54.62% (either) or 46.92% (both) accuracy. A plausible cause for the classifier’s failure in this respect is that one subject’s signal may be drowned out by the other’s. An advanced mathematical model needs to be developed to address this problem.

#### Long short-term memory (LSTM)

LSTM is Recurrent Neural Network’s popular architecture for dynamical systems and real-time temporal signal processing. Its robustness is proven in speech recognition and intrusion detection applications. By letting the output serve as its own input, the recurrent layer creates, in effect, a feedback loop or “memory” to track intrinsic dependencies during dataflow. Furthermore, the memory is controlled in the location of being received (input gate), retained (forget gate), or used in the output (output gate) by relying on nonlinear functions performing as regulators. In this work, inputs of the LSTM are two-dimensional array, which consist of raw data harnessed by five fiber sensors. The LSTM layer has 100 hidden units. The output of the LSTM layer is the final output of each unit. A fully connected layer following the LSTM layer transform the output from these 100 hidden units into 8 features. A softmax layer transforms these 8 features into the classification result (among 8 categories). In regard to data generated by eight people running, a comparison is made between CNN and LSTM (see Table 3). LSTM’s low accuracy in distinguishing among the eight people reveals its weakness in segmented signal recognition. With limited operational capability in terms of computer-based data acquisition/processing, LSTM is neither competent nor adequate for completing the longstanding process of acoustic signal extraction. Furthermore, its analysis of segmented temporal data is inferior to feature recognition in the frequency domain in this application.

**Table 3:** Classification results using different machine learning algorithms or neural networks.

Method	Accuracy (one person walking)	Accuracy (one person running)
CNN	78.75–86.25%	76.25–86.25%
LSTM	12.50–20.62%	7.69–16.35%
ECOC	63.44–76.92%	69.23–80.77%
KNN	44.69–62.50%	48.08–61.54%
NB	42.50–64.42%	50.96–59.62%

#### Error-correcting output codes (ECOC), K-nearest neighbor (KNN), and Naïve Bayes (NB) classifiers

Three more methods were introduced to examine the robustness in the frequency domain: the ECOC, KNN, and NB classifiers. These methods provide strong outcomes in a variety of applications^31^. There are well-developed and fully integrated libraries and toolboxes for these methods in most scientific programming languages, including MATLAB. ECOC is a multi-class extension of the SVM algorithm which combines lots of binary classifiers to solve multi-class problems. One-vs-one coding was used in this experiment, which brought 28 binary classifiers to distinguish eight participants as eight classes. Each binary classifier was a linear SVM that used a box constraint of 1 (a parameter which prevents overfitting from outlier). The KNN classifier is an instance-based supervised classification method. It classifies the test sample to the class to which a plurality of its *k* nearest neighbors belong. The distance function *k* was optimized to be set as 1. It simply assigned the test sample to the class of its nearest neighbor. NB classifier is a probabilistic classification method based on Bayesian inference. It assumes the statistic independence of each component of the input variable. Given the training data, a Gaussian probability model with empirical prior was learned for each class. The test samples were classified into multiple classes corresponding to the maximum posteriori estimate of the model parameters. Detailed descriptions on these three frequency-domain machine learning algorithms can be found in Ref.^31^.

Table 3 presents the accuracies to classify one person walking and running from eight participants by using various machine learnings. Among all machine learning algorithms tested in this work, CNN produces best outcomes. This is consistent with other works where CNN yielded better results for spectrum analysis^32,33^. Machine learning algorithms often have strong assumptions about the statistic natures of the data. For instance, NB has a strong assumption about the distribution of data, and KNN assumes that the data clusters with respect to a given metric. The assumptions behind CNN are relatively generic, and it has in addition benefits of its depth, which hierarchically extracts features for the data. This might be the reason for higher classification accuracy than other approaches.

### Unsupervised machine learning

Unlike supervised learning, unsupervised learning methods extract features from the training data without the use of any labels. This eliminates the tedious work of labeling a large amount of training data needed for supervised learning schemes. Clustering is an example of unsupervised learning by partitioning the data into different clusters based on certain criteria. K-means clustering with Euclidean distance is a basic clustering problem commonly used for feature extraction. Its objective is to minimize the sum of total variances within the clusters. While the exact solution of K-means clustering is NP-hard, Lloyd's algorithm provides an effective method of relaxation by iteratively updating the centroids of the clusters.

Let ${\left({x}_{i}\right)}_{1\le i\le N}$ be the datapoints. Denote the centroids of cluster $k$ at iteration $j$ by ${c}_{kj}$. Lloyd's algorithm has two steps in each iteration: (1) classify each datapoint ${x}_{i}$ into the cluster whose centroid is closest to ${x}_{i}$ (i.e., $\mathrm{arg}\underset{k}{\mathrm{min}}\Vert {x}_{i}-{c}_{kj}\Vert$); (2) calculate the centroids for the next iteration based on the current clustering

3 $${c}_{k\left(j+1\right)}=\frac{1}{\left|{C}_{kj}\right|}{\sum }_{i\in {C}_{kj}}{x}_{i}$$

where ${C}_{kj}$ is set of the indices of all datapoints in cluster $k$ at iteration $j$. Despite its efficiency for well-behaved datasets, Lloyd's algorithm may show a slow convergency and does not guarantee the optimality of the solution. The parameter *K* also greatly impacts the clustering results.

One of the difficulties of unsupervised learning is the interpretation of results. This is because the K-means algorithm itself does not label the clusters. For that reason, we proceeded with a hierarchical two-class clustering, and at each step we compared the result with potential features. Instead of directly predict the precise category a sample belongs to, a hierarchical two-class clustering makes several different binary classifications between two larger categories in sequence. For instance, the algorithm decides first whether the sample is a running sample or a walking sample. If it’s a running sample, then it decides which direction the subject is running. This approach is more suitable for unsupervised learning, as it may be unknown to the algorithm how many precise categories there are. It should be mentioned that the accuracy resulting from the comparison is not an objective evaluation of the unsupervised learning method, but simply an indication of the type of features the algorithm prioritizes and finds. When the algorithm makes unsupervised binary classifications, human operators don’t know which criteria these classifications are based on, so classifications made by the algorithm must be combined with the known characteristics of the data in order to understand whether a classification according to the type, or direction, or location of the motion. These comparisons and the associated accuracies are shown in Table 4.

**Table 4:** Classification results using K-means clustering for various scenarios.

Training	Top clusters	Accuracy
One person running	Direction1	53.85% (all)
	Direction2	92.31% (individual)
Unidirectional, one person running	Person	77.65% (all)
		100% (individual)
One person walking	Direction1	65.38% (all)
	Direction2	96.15% (individual)
Unidirectional, one person walking	Person	88.46% (all)
		94.23% (individual)
One person walking + one person running	Movement1 (running) Movement2 (walking)	94.71%

Table 4 summarizes the results from implementing K-means clustering. The range of uncertainty was not investigated, since unsupervised machine learning finds relations inside the data itself and organizes them into several clusters. Therefore, they were not divided into training and validation groups. The top cluster depends on the subject and how the data are fed to the algorithm. As in direction recognition, if data for each subject are fed individually, the accuracy reaches 92.31%. If the data of all eight subjects are fed together, the accuracy drops as low as 53.85% for certain subjects. This can be explained by the fact that more subjects in the input data equal more secondary features in the dataset—a potential source of confusion for the algorithm. Precise and successful identification must result from sifting through a multitude of possibilities and determining a specific combination of features, such as the strength, duration, and location of steps, as well as the speed and rhythm of movement. However, unsupervised learning only extracts those features it deems most important. Without deterministic guidance, some covert—and possibly useful—characteristics may be lost. As a result, the accuracy of the algorithm is highly variable in both human identification and direction of movement.

Unsupervised learning normally has poor performance than supervised learning, which is consistent with results summarized in Table 4. However, in some applications, supervised learning is inaccessible due to the lack of labeled samples for training. Therefore, studies of unsupervised learning for human locomotion identification can be very meaningful as it could reveals level of performance of machine learning algorithms without much prior knowledge of dataset. These results are not simply replaceable by supervised learning studies.

## Conclusion

This paper describes a bottom-up approach to improve efficacy of machine learning algorithm. Through SNR improvement of distributed fiber sensors, high quality and high spatial resolution data can be harnessed to provide better training of machine learning algorithm to identify subtle difference existed in datasets. Artificial Rayleigh scattering features were induced to enhance weak intrinsic Rayleigh scattering in single-mode fibers by more than 35 dB through femtosecond laser direct writing schemes. This led to improved SNR and sensitivity for distributed fiber acoustic sensors, thus generating high-quality acoustic data carrying characteristics of human locomotion. Such large amount acoustic data was used to train various machine learning algorithms, bringing higher accuracy in identifying individuals and various events. Machine learning is a powerful tool in PR, proven to manifest covert features used in classification. It meets the requirements for both human identification and locomotion recognition, with an accuracy of over 76.25% when using supervised deep neural networks and 77.65% when using unsupervised machine learning algorithms. Despite these strides, the potency of artificial intelligence algorithms relies on both the quantity and quality of training data. This paper gives a detailed example of how high spatial resolution and bandwidth data from distributed fiber sensors can be very valuable and new stream of data sources for artificial intelligence.
